# Long Non-Coding RNAs in Ovarian Cancer: Mechanistic Insights and Clinical Applications

**DOI:** 10.3390/cancers17030472

**Published:** 2025-01-30

**Authors:** Sneha Basu, Revathy Nadhan, Danny N. Dhanasekaran

**Affiliations:** 1 Stephenson Cancer Center, The University of Oklahoma Health Sciences Center, Oklahoma City, OK 73104, USA; sneha-basu@ouhsc.edu (S.B.); revathy-nadhan@ouhsc.edu (R.N.); 2 Department of Pathology, The University of Oklahoma Health Sciences Center, Oklahoma City, OK 73104, USA; 3 Department of Cell Biology, The University of Oklahoma Health Sciences Center, Oklahoma City, OK 73104, USA

**Keywords:** ovarian cancer, long non-coding RNA (lncRNA), biomarkers, therapeutic targets, cancer pathogenesis, chemoresistance, tumor microenvironment, epigenetic regulations, gene expression, personalized medicine

## Abstract

**Background/Objectives:** Ovarian cancer is a leading cause of gynecological cancer mortality worldwide, often diagnosed at advanced stages due to vague symptoms and the lack of effective early detection methods. Long non-coding RNAs (lncRNAs) have emerged as key regulators in cancer biology, influencing cellular processes such as proliferation, apoptosis, and chemoresistance. This review explores the multifaceted roles of lncRNAs in ovarian cancer pathogenesis and their potential as biomarkers and therapeutic targets. **Methods:** A comprehensive literature review was conducted to analyze the structural and functional characteristics of lncRNAs and their contributions to ovarian cancer biology. This includes their regulatory mechanisms, interactions with signaling pathways, and implications for therapeutic resistance. Advanced bioinformatics and omics approaches were also evaluated for their potential in lncRNA research. **Results:** The review highlights the dual role of lncRNAs as oncogenes and tumor suppressors, modulating processes such as cell proliferation, invasion, and angiogenesis. Specific lncRNAs, such as HOTAIR and GAS5, demonstrate significant potential as diagnostic biomarkers and therapeutic targets. Emerging technologies, such as single-cell sequencing, provide valuable insights into the tumor microenvironment and the heterogeneity of lncRNA expression. **Conclusions:** LncRNAs hold transformative potential in advancing ovarian cancer diagnosis, prognosis, and treatment. Targeting lncRNAs or their associated pathways offers promising strategies to overcome therapy resistance and enhance personalized medicine. Continued research integrating omics and bioinformatics will be essential to unlock the full clinical potential of lncRNAs in ovarian cancer management.

## Simple Summary

Ovarian cancer is a highly fatal disease, often diagnosed at advanced stages due to non-specific symptoms. This review focuses on the role of long non-coding RNAs (lncRNAs) in ovarian cancer, highlighting their dual nature as oncogenes or tumor suppressors. These molecules regulate critical cancer processes, including cell growth, survival, metastasis, and therapy resistance. By exploring their complex structures and functions, this research emphasizes the potential of lncRNAs as diagnostic markers, prognostic indicators, and therapeutic targets. Integrating insights from cutting-edge technologies like bioinformatics and single-cell sequencing, this review aims to provide a comprehensive understanding of lncRNAs in ovarian cancer. The findings could significantly advance personalized cancer medicine, offering new strategies to improve detection, treatment, and patient outcomes.

## 1. Introduction

Ovarian cancer (OC) is recognized as the eighth most common cancer among women worldwide and a leading cause of female mortality, with approximately 207,000 deaths annually [1,2]. Globally, 1 in 70 women are diagnosed with OC, and over 60% of these cases result in mortality [3]. According to GLOBOCAN cancer data, the annual incidence of OC is projected to rise to approximately half a million by 2050, with the highest increases expected in Africa and Asia [4,5]. In the United States, it ranks fifth in cancer-related deaths amongst women with a predicted 611,720 OC-associated deaths for the year 2024 [4]. Predominantly originating from epithelial cells, OC presents significant challenges for early detection due to its non-specific symptoms [6,7], leading to its description as a “silent killer” [8]. The consequent late-stage diagnoses severely limit therapeutic options and contribute to poor patient outcomes. These outcomes are further complicated by the heterogeneity of OC, the lack of effective targeted therapy, and the development of resistance to existing therapies [9,10,11]. These challenges underscore the urgent need for newer personalized therapeutic strategies.

Long non-coding RNAs (lncRNAs), RNA molecules consisting of over 200 nucleotides that do not code for proteins, have emerged as critical players in the development and progression of various cancers, including OC [12,13,14]. Acting as oncogenes or tumor suppressors, lncRNAs are involved in a wide array of cellular processes, such as gene regulation, mRNA stabilization, molecular scaffolding, post-translational modifications, epigenetic regulation, and chromatin remodeling [15,16,17]. Their dysregulation is linked to the hallmarks of cancer including cellular proliferation, invasion, metastasis, resistance to chemotherapy, angiogenesis, cancer stemness, and alterations in epigenetic and transcriptional landscapes [13,14,18]. In OC, the aberrant expression of lncRNAs contributes to the disease’s aggressive nature, making them promising candidates as biomarkers and targets of therapy [19,20].

This review explores the multifunctional role of lncRNAs in OC, providing an in-depth analysis of how they regulate various cellular processes and contribute to OC pathogenesis. In addition, a detailed examination of lncRNAs as a novel class of signaling molecules, diagnostic and prognostic biomarkers, and potential therapeutic targets in OC is discussed, emphasizing their potential in early detection, prognosis, and treatment.

## 2. LncRNAs: Structure and Function

**Figure 1:**
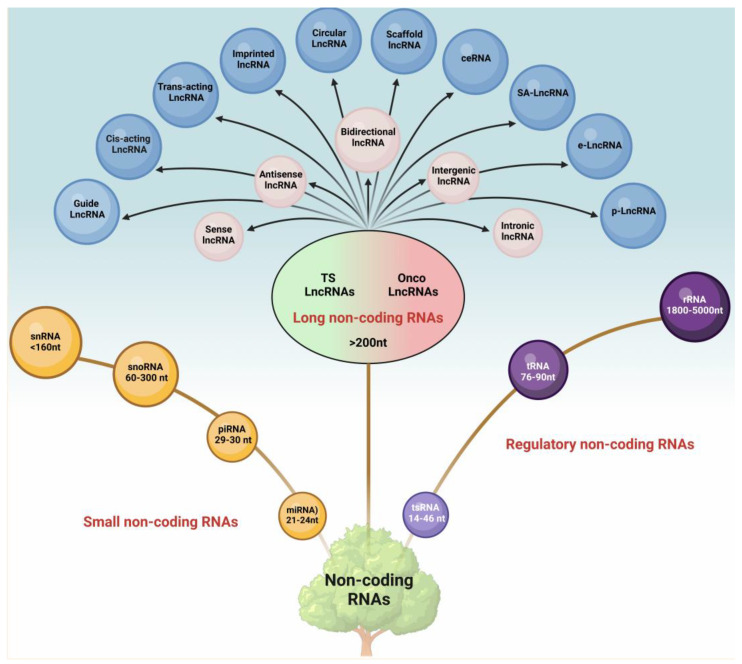
Classification tree for non-coding RNAs. Non-coding RNAs can be classified into three major groups, namely the small non-coding RNAs that are less than 200 nt in length, the long non-coding RNAs (lncRNAs) that are greater than 200 nt in length, and the specific subclass of regulatory non-coding RNAs. The smaller non-coding RNAs include the miRNA, piRNA, snoRNA, and other snRNAs. The regulatory non-coding RNAs encompasses the tsRNA, tRNA, and rRNA. The lncRNAs can be classified into sense, antisense, bidirectional, intergenic, and intronic based on their position in the genome. Based on the functional classification, lncRNAs can be either guide, cis-/trans-acting, imprinted, scaffold, circular, competing endogenous, spliceosome-associated, enhancer-associated, or promoter-associated lncRNAs. (ceRNA: competing endogenous lncRNA; e-lncRNA: enhancer-associated lncRNA; miRNA: microRNA; onco-lncRNA: oncogenic lncRNA; p-lncRNA: promoter-associated lncRNA; piRNA: piwi interacting RNA; rRNA: ribosomal RNA; SA-lncRNA: spliceosome-associated lncRNA; snRNA: small nuclear RNA; snoRNA: small nucleolar RNA; tRNA: transfer RNA; tsRNA: transfer RNA derived small RNAs; TS-lncRNA: tumor-suppressor lncRNA).

The superfamily of non-coding RNAs (ncRNAs) encompasses a diverse range of RNA molecules that do not code for proteins but play critical regulatory and structural roles in cellular processes [15,16]. This superfamily is broadly categorized into small ncRNAs, typically less than 200 nucleotides in length, and long ncRNAs (lncRNAs), which are greater than 200 nucleotides and housekeeping or regulatory ncRNAs (Figure 1).

Small ncRNAs comprise microRNAs (miRNAs, 21–24 nt), piwi-interacting RNAs (piRNAs, 29–30 nt), small nuclear RNAs (snRNAs, <160 nt), and small nucleolar RNAs (snoRNAs, 60–300 nt). Regulatory ncRNAs represent another distinct group, encompassing transfer RNAs (tRNAs, 76–90 nt), transfer RNA-derived small RNAs (tsRNAs, 14–46 nt), and ribosomal RNAs (rRNAs, 1800–5000 nt).

LncRNAs, a highly diverse subset of ncRNAs, are further classified based on their genomic location, structure, and function. Functionally, lncRNAs are often categorized into tumor-suppressor and oncogenic lncRNAs, highlighting their pivotal roles in cancer development and progression. Despite not being translated, the ncRNAs play a significant role in regulating gene expression and various cellular processes [21,22]. LncRNAs are one of the major classes of ncRNAs involved in the regulation of chromosomal remodeling, transcription, post-transcriptional modifications, and diverse cell signaling pathways [23,24]. They also modulate the sub-cellular localization of different proteins and RNAs within the cytoplasm [15,17,25]. The aberrant and asynchronous expression of lncRNAs is linked to a variety of cancers, including OC, making them potential biomarkers [26]. The functions and localization of lncRNAs, whether in the nucleus or cytoplasm, are highly tissue-specific and are influenced by their complex structures [27,28].

### 2.1. Primary, Secondary, and Tertiary Structures of lncRNAs

**Figure 2:**
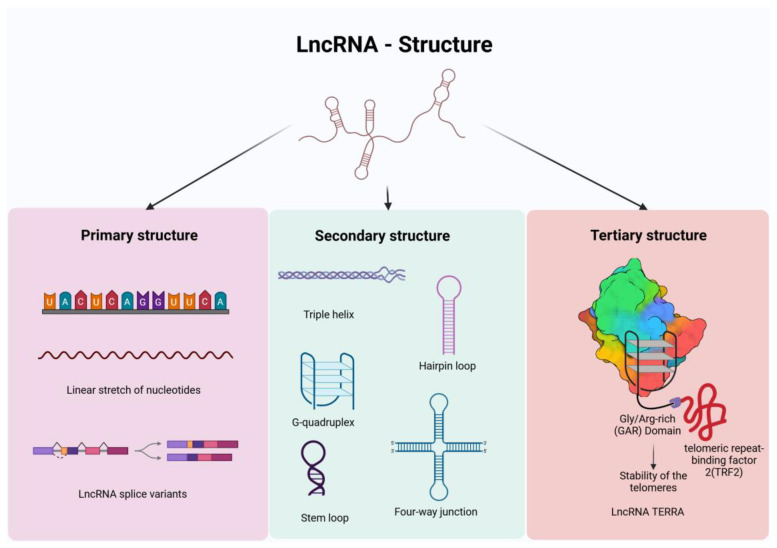
Structural differences in lncRNAs across the three levels: primary, secondary, and tertiary. The primary structure of lncRNA includes a linear stretch of nucleotides and splice variants of the same lncRNA, showcasing sequence diversity. The secondary structure includes a triple helix, hairpin loop, G-quadruplex, stem-loop, and four-way junction demonstrating the complex folding patterns of the primary lncRNA. The tertiary structure is depicted with an example: the lncRNA TERRA, which forms a complex G-quadruplex to interact with the telomeric-repeat binding factor 2 (TRF2) to confer stability to the telomeres.

The regulatory capabilities of lncRNAs can be attributed to their structural configurations encompassing primary, secondary, and tertiary structures (Figure 2).

#### 2.1.1. Primary Structure

The primary structure of lncRNAs is a linear sequence of nucleotides, namely adenine, guanine, cytosine, and uracil. Typically transcribed by RNA Polymerase II, lncRNAs may sometimes be transcribed by RNA Polymerase III [29]. They often have multiple splice variants, leading to diverse transcripts with varying lengths and functions. Unlike protein-coding genes, lncRNAs generally exhibit poor evolutionary sequence conservation across species, although some conserved regions exist [30,31]. This primary sequence forms the backbone of the lncRNA, determining its basic properties and serving as the foundation for the more complex secondary and tertiary structures that dictate its functionality [13].

#### 2.1.2. Secondary Structure

Secondary structures in lncRNAs arise from the folding and interactions among base pairs within the primary sequence through hydrogen bonds, forming structural elements such as pseudoknots, stem-loops, hairpins, triple helix structures, and kissing loop interactions [13,32]. These structures confer stability and provide a structural basis for the diverse functions of the lncRNAs [22,33]. For example, MALAT1, known for its role in the regulation of gene expression, forms a triple helix structure, which protects it from 3′-5′ exonucleases [34]. Similarly, a specific 24-nucleotide conserved region in the lncRNA XIST forms a tetraloop structure, a stem-loop variant that involves four nucleotides for the formation of the loop, which allows XIST to interact with YTH N6-Methyladenosine RNA Binding Protein C1 (YTHDC1), facilitating XIST-mediated transcription silencing [35]. In fact, it has been inferred that XIST recruits over 80 distinct proteins through its structural motifs, enabling the efficient silencing of the X-chromosome through multiple mechanisms [36]. Certain lncRNAs such as Growth Arrest-Specific 5 (GAS5) exhibit multiple stable secondary structure conformations including a 3′-serine and arginine (3′ SR)-binding short hairpin motif that acts as a scaffold to interact with steroid receptors to inhibit steroid-mediated cancer cell growth and survival [37].

#### 2.1.3. Tertiary Structure

The tertiary structures refer to the three-dimensional conformations of lncRNAs formed by further folding and interactions among secondary structural elements formed by both the classical Watson and Crick base pairing and the non-canonical interactions [18]. The resulting higher-order structures such as G-quadruplexes—which involve guanine-rich regions folding to form a stable, four-stranded structure—form specific docking sites for interactions with proteins, RNA, or DNA [33]. This is exemplified in the case of the lncRNA HOTAIR, which relies on a G-quadruplex structure for its interaction with Polycomb Repressive Complex 2 (PRC2) proteins in the epigenetic silencing of genes [38]. Likewise, the G-quadruplex motifs of MALAT1 are essential for its binding with the non-POU Domain-containing Octamer-Binding (NONO) protein, a canonical nuclear paraspeckle protein [39]. Similarly, NEAT1 forms a G-quadruplex, essential for the formation of paraspeckles nuclear bodies, through the interaction with NONO and splicing factor proline and glutamine-rich (SPFQ) proteins [40,41,42]. In a similar vein, the complex stem-loop structures and G-quadruplex motifs of the lncRNA TERRA are essential for the regulation of the telomere function, which is often altered in OC [43,44]. Tertiary structures also contribute significantly to the stability of lncRNAs, protecting them from rapid degradation. This stability is crucial for lncRNAs to sustain their regulatory roles in eliciting specific cellular responses [45].

The intricate architecture of lncRNAs, spanning from basic primary sequences to complex tertiary structures enable lncRNA to participate in a broad spectrum of biological functions, from the regulation of gene expression to the maintenance of chromatin architecture.

### 2.2. Positional Diversity of lncRNAs

**Figure 3:**
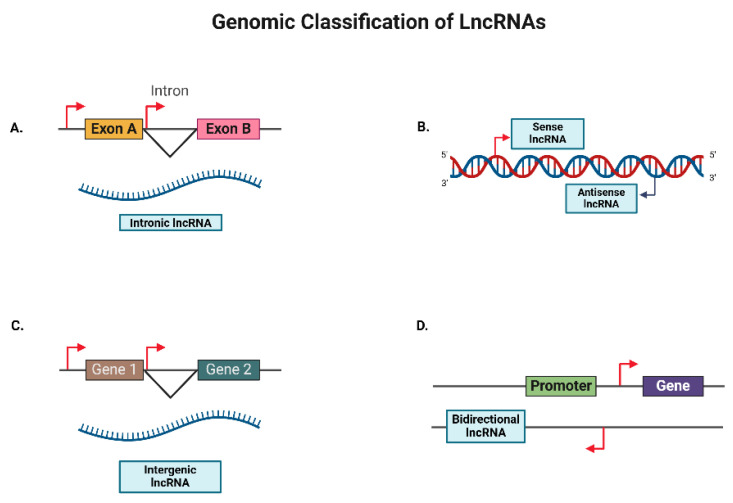
Genomic classifications of lncRNAs. LncRNAs can be classified on the basis of their origin in the genome. (**A**) Intronic LncRNA: transcribed from the intronic region of a transcript; (**B**) Sense lncRNA and antisense lncRNA: transcribed from the sense and antisense strand, respectively; (**C**) Intergenic lncRNA: transcribed from the region between two genes; (**D**) Bidirectional lncRNA: transcribed from the promoters of coding genes but in an opposite direction. (LncRNAs—long non-coding RNAs).

LncRNAs can be classified into various categories based on their genomic location, including intronic, antisense, sense, intergenic, and bidirectional, each with a distinct mode of action (Figure 3) [46].

#### 2.2.1. Intronic LncRNAs

Intronic lncRNAs are transcribed from the introns (non-coding regions) of coding genes. They often regulate the expression of gene from which they originate [15]. For example, the lncRNA SPRY4-IT1, originating from the *SPRY4* gene’s intronic region, suppresses the expression of SPRY4, thus inhibiting epithelial–mesenchymal transition (EMT) and metastasis in OC [47].

#### 2.2.2. Antisense LncRNAs

Antisense lncRNAs are complementary to the protein-coding gene and are transcribed from the opposite strand of coding sequences. They often regulate the expression of the cognate sense mRNAs [48]. An example here is CDKN2B-AS1 (ANRIL), an antisense lncRNA of the *CDKN2B* gene, which is overexpressed in the OC cell lines SKOV3, A2780, and HO-8910. ANRIL suppresses CDKN2A and CDKN2B expression to promote OC cell migration and invasion [49]. In some instances, antisense lncRNAs such as RUNX1-IT1, transcribed from the same promoter as the *RUNX1* gene, does not suppress its expression. RUNX1-IT1 promotes EMT in OC by acting as a scaffold linking STAT1 and the NuRD complex, eventually leading to the activation of NF-kB [50]. Therefore, it is not necessary that an antisense lncRNA will act only on its adjacent gene; it can also have different regulatory mechanisms.

#### 2.2.3. Intergenic LncRNAs

Intergenic lncRNAs are located between the coding genes. They have been shown to modulate the expression of nearby as well as distal genes through diverse regulatory mechanisms [51]. One example involves the regulation of gene expression by the lncRNA GAS5, an intergenic tumor-suppressor lncRNA located on chromosome 1 [52,53]. GAS5 has been shown to regulate the expression of the *PARP1* gene, which is also present on chromosome 1 by recruiting the transcription factor E2F4 to its promoter, so as to increase the chemosensitivity in a panel of OC cell lines [54]. It has also been shown that GAS5 can mediate tumor-suppressor function by inhibiting the expression levels of the distal gene *hnRNPK1,* thereby inhibiting the AKT pathway [55] and/or promoting the expression of the pro-apoptotic Bcl-2 family proteins BAK and BAX in OC cells [56].

#### 2.2.4. Bidirectional LncRNAs

Bidirectional lncRNAs are transcribed in the opposite directions of an adjacent coding gene, often sharing a bidirectional promoter and regulatory elements. For instance, the lncRNA known as ncRNA-RBI/RB1-DT/LINC00441 is a bidirectional RNA that is transcribed from the promoter of the retinoblastoma gene *RB1* [57], and it facilitates the expression and translocation of calreticulin (CALR) from the endoplasmic reticulum to the cell surface, initiating an anticancer immune response [57].

### 2.3. Functional Diversity of LncRNAs

**Figure 4:**
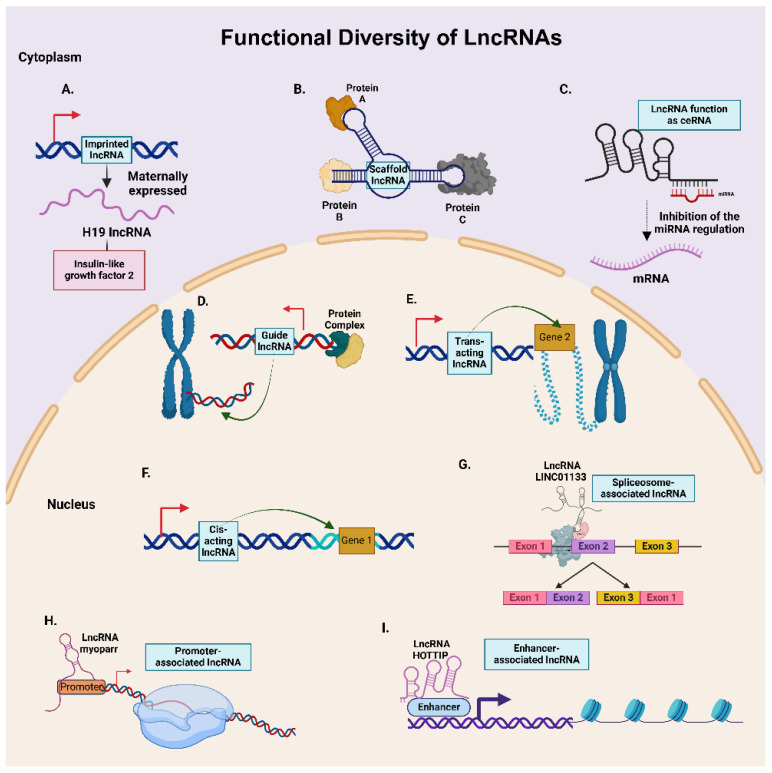
Functional diversity of lncRNAs. In the cytoplasm, lncRNAs function as (**A**) imprinted lncRNAs, (**B**) scaffold lncRNAs, and (**C**) ceRNA, showcasing their diverse regulatory mechanisms throughout the cell. Within the nucleus, the lncRNAs can (**D**) act as a guide lncRNA, function either as (**E**) trans- or (**F**) cis-regulatory elements, or can be associated with (**G**) the spliceosome complex, (**H**) promoters, or (**I**) enhancers.

LncRNAs, with their structural and positional diversity, exhibit a remarkable functional diversity that allows them to play complex regulatory roles in modulating a wide array of oncogenic responses (Figure 4). The functional classification of lncRNAs includes the following categories.

#### 2.3.1. Cis- and Trans-Acting LncRNAs

Cis-acting lncRNAs regulate genes on the same chromosome, often through chromatin modification, enhancer-like activity, and transcriptional interference [58]. For example, the lncRNA KCNQ1OT1, located at chromosome 11p15.5, regulates nearby genes like *SLC22A18* and *CDKN1C*, both of which located are located in a nearby genomic location in Chr. 11p15.4, through histone methylation to suppress their expressions [59].

Trans-acting lncRNAs influence distally located genes, modulating cellular processes by sequestering miRNAs, interacting with RNA–protein complexes, or altering chromatin structure [58]. For instance, RHPN1-AS1 promotes OC cell proliferation by sequestering miR-1299 [57]. In another example, RUNX-IT1 acts as a scaffold for the NuRD complex and STAT1 which allows the recruitment of several histone modifiers, eventually activating NF-kB and promoting OC [50].

#### 2.3.2. Enhancer and Promoter-Associated LncRNAs

Enhancer-associated lncRNAs are transcribed from enhancer regions of the gene and modulate gene expression by influencing the activity of enhancers of the cognate gene [15]. An example is the lncRNA HOTTIP, transcribed from the 5′ end of the *HOXA* gene cluster enhancer, which regulates *HOXA* gene expression by binding to the WDR5 adaptor protein, facilitating H3 lysine 4 trimethylation [60].

Promoter-associated lncRNAs are transcribed from the promoter regions of coding genes and regulate the transcription of these genes. For example, the lncRNA FOXCUT is transcribed from the promoter region of the *FOXC1* gene and regulates its expression, essential for cancer progression in esophageal cancer [57]. Some promoter-associated lncRNAs act as scaffolds for assembling transcriptional regulatory complexes, affecting the transcription of specific genes. The lncRNA KHPS1, transcribed from the *SPHK1-C* gene promoter, forms a triplex RNA–DNA structure, recruiting transcription factors and activating SPHK1 expression [61].

#### 2.3.3. Competing Endogenous RNAs (ceRNAs)

Certain lncRNAs act as ceRNAs, sequestering miRNAs to modulate the gene expression of other coding genes [62]. For example, the lncRNA UCA1, which is identified as an LPA-stimulated lncRNA, sequesters the let-7 family of miRNAs, promoting OC progression by suppressing the tumor-suppressor roles of let-7 miRNAs [63]. The lncRNA LINC01123 contributes to increased angiogenesis in OC by sequestering miR-516b-5p and upregulating vascular endothelial growth factor, VEGFA, expression [64].

#### 2.3.4. Guide and Scaffold LncRNAs

Guide lncRNAs recruit regulatory proteins to the target regions of the chromatin or DNA to regulate gene expression [65]. UCA1, acting as a guide lncRNA, promotes YAP dephosphorylation and increased expression of its target genes in OC cell lines through its binding to AMOT, which guides and stimulates its interaction with YAP [66].

In a variation on the theme, scaffold lncRNAs facilitate the assembly of various proteins and RNAs to form functional ribonucleoprotein (RNP) complexes [22]. LINC00176 functions as a molecular scaffold, facilitating the interaction between BCL3 and p50 which binds to the promoter region of the ceruloplasmin, *Cp*, gene, thereby activating its expression and promoting oncogenic pathways in OC cell lines via the NF-κB signaling pathway [67].

#### 2.3.5. Imprinted LncRNAs

Imprinted lncRNAs are associated with genomic imprinting, expressing only one allele of paternal or maternal origin [68]. A well-studied lncRNA is H19, which is maternally expressed and found at high levels in the carboplatin-resistant OC cell line SKOV3 compared to its non-resistant counterparts. H19 acts as a ceRNA for miR-29b-3p and confers carboplatin resistance through the upregulation of drug-resistance-associated proteins MRP1, P-gp, and LRP and STAT3, which are the downstream targets of miR-29b-3p [69,70].

#### 2.3.6. Spliceosome-Associated LncRNAs

LncRNAs associated with alternative splicing can regulate this process either by directly interacting with spliceosome components as scaffolds (spliceosome-associated lncRNAs) or by indirectly modulating the expression of spliceosomal components, thereby influencing alternative splicing and differential gene expression [71,72]. For instance, in colorectal cancer, LINC01133 directly binds to the splicing factor SRSF6. Overexpression of LINC01133 inhibits invasion and migration in colorectal cancer by restricting the activity of SRSF6 [73]. An example of indirect regulation is provided by UCA1, which sequesters miR-184, resulting in increased levels of SF1, a key factor in spliceosomal assembly. Elevated UCA1 expression confers cisplatin resistance in oral squamous cell carcinoma by regulating SF1 levels [74].

Although no spliceosome-associated lncRNAs have been reported in OC to date, active research is underway to assess the role of lncRNAs in modulating alternative splicing by binding directly to splicing factors. Unpublished data from our group demonstrate the role of UCA1 as a spliceosome-associated lncRNA in regulating alternative splicing in OC. There has been a study demonstrating the indirect modulation of splicing by the lncRNA MALAT1 in OC [75]. MALAT1 has been shown to regulate the expression of several splicing regulatory (SR) proteins, including RNA-binding protein fox-1 homolog 2 (RBFOX2), which governs alternative splicing events in HEY and OVCA420 OC cells. This activity is correlated with the decreased expression of the pro-apoptotic splice variant Kinesin Family Member 1Bb (KIF1Bb) protein along with the increased expression of the non-apoptotic truncated splice variant KIF1Ba, both of which promote OC cell proliferation, invasion, and anchorage-independent growth [75].

The remarkable structural and functional diversity of lncRNAs enables them to play critical roles in OC, impacting on cellular events ranging from chromatin remodeling to immune response modulation. Functional diversity, along with context-dependency, is crucial in many cancers, including OC. For instance, factors such as tumor hypoxia can alter the expression of specific lncRNAs and their target genes, directing lncRNAs toward specific functional responses [76]. Aberrant expression of both oncogenic and tumor-suppressor lncRNAs contributes to the genesis and progression of OC, with different lncRNAs playing distinct roles in either promoting or inhibiting cancer development [77]. The following section explores how these lncRNAs contribute to the development and progression of OC, emphasizing their roles as both oncogenic drivers and tumor suppressors.

## 3. LncRNAs in Ovarian Cancer

**Figure 5:**
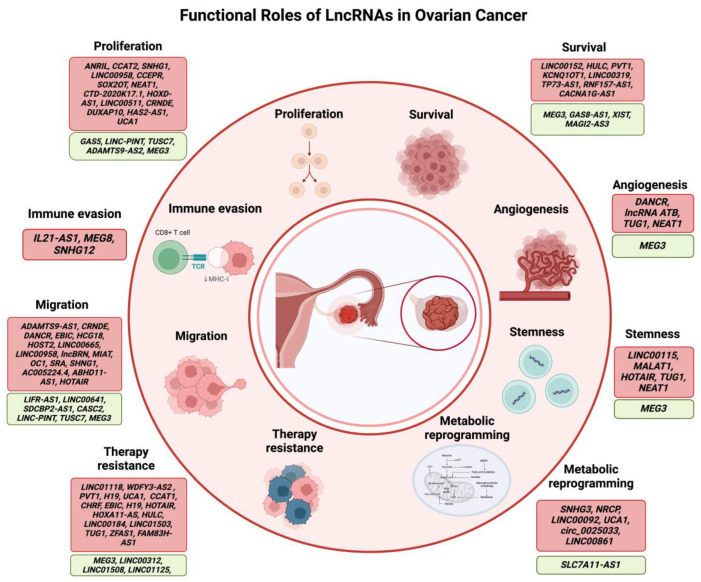
Functional roles of lncRNAs in ovarian cancer. LncRNAs are critically involved in cell proliferation, cell survival, angiogenesis, stemness, metabolic reprogramming, therapy resistance, cell migration, and, immune evasion in ovarian cancer. Examples of lncRNAs that induce these processes and act as oncogenes in OC are highlighted in red boxes, whereas those that tend to reduce these processes are highlighted in green boxes.

LncRNAs play diverse and crucial roles in gene expression, chromatin remodeling, and post-translational regulation, significantly impacting OC [78]. Acting as either oncogenes or tumor suppressors, lncRNAs are often differentially expressed in OC cells compared to non-cancerous cells [79], engaging in complex regulatory mechanisms essential for OC progression (Figure 5).

The discovery of their involvement in oncogenic pathways has spurred extensive research into the specific functions of lncRNAs in OC. Despite the vast number of lncRNAs expressed, only a small fraction have been characterized, with even fewer being definitively linked to OC. Table 1 and Table 2 provide a summary of the key lncRNAs identified as oncogenes and tumor suppressors in OC, respectively. These identified lncRNAs have become the focus of detailed studies aimed at elucidating their roles in OC genesis and progression.

### 3.1. LncRNAs in Ovarian Cancer Cell Proliferation

OC cells, like other cancer cells, promote cancer growth by increasing cellular proliferation through dysregulated cell cycle machinery [170]. LncRNAs play diverse roles in this process, influencing key events such as cell cycle progression, resistance to apoptosis, and overall cellular proliferation.

#### 3.1.1. Regulation of Cell Proliferation

Abnormal expression of some lncRNAs activates specific signaling pathways, creating favorable conditions for cancer cells to proliferate. The lncRNA Colon cancer-associated Transcript 2 (CCAT2) has been shown to promote cell proliferation through the Wnt/β-catenin pathway in the OC cell lines SKOV3 and A2780. Inhibition of the Wnt/β-catenin pathway prevents these cells from progressing from the G1 to the S phase. In addition, the silencing of CCAT2 reduces the expression of c-MYC and MMP-7, which are critically involved in cell proliferation and metastasis [171]. Likewise, the lncRNAs SNHG1, LINC00958, CCEPR, and HOXD-AS1 activate the Wnt/β-catenin pathway, positively regulating OC cell proliferation [89,105,123,136]. The lncRNA NEAT1, stabilized by the protein LIN28, promotes cellular proliferation in vivo which was detected by an increase in expression of Ki67, an essential proliferation index marker. Additionally, NEAT1 promotes metastasis in OVCAR3 and A2780 OC cell lines by sequestering miR-506, which positively regulates the expression of vimentin, ZEB1, and Snail2. However, the interaction of NEAT1 with both LIN28 and miR-506 has not been completely elucidated [172]. In another study, NEAT1 has been reported to promote proliferation in OC by acting as a ceRNA for miR-365 resulting in the upregulation of Fibroblast growth factor 9 (FGF9). FGF9 is a positive regulator of proliferation and angiogenesis in multiple cancer types. However, analysis of specific downstream effectors of FGF9 in OC requires further studies [173]. The lncRNA CTD-2020K17.1 is highly upregulated in metastatic tissue and OC cell lines. SKOV3 cells highly expressing the lncRNA CTD-2020K17.1 are actively dividing, and their distribution is more in the S phase. CTD-2020K17.1 has been associated with the Caspase-associated recruitment domain (CARD11), a positive regulator of the NF-κB pathway [174].

#### 3.1.2. Regulation of Cell Cycle Progression

When lncRNAs interfere with cyclin-dependent kinases (CDKs), cyclin-dependent inhibitors (CDIs), and cyclins, they disrupt the mechanisms of cell cycle arrest, leading to uncontrolled cell proliferation. This interference disables the normal checks and balances that prevent excessive cell division. For instance, the lncRNA ANRIL is upregulated in OC patient tissues, contributing to cellular proliferation by increasing Bcl-2 and downregulating the CDK-inhibitors p15INK4a and p15INK4b. Knockdown of ANRIL restricts cell cycle transition in the S phase, inhibiting cell proliferation [175]. Another lncRNA, UCA1, acts as an oncogene in SKOV3 and OVCAR8 OC cell lines by modulating the expression of the essential cell cycle regulators CCND1, CCND3, and Ki67, eventually promoting cell proliferation [63]. The lncRNA SOX2OT is highly upregulated in the HO-8910PM OC cell line and its knockdown restricts the cell cycle progression in the G0/G1 phase and suppresses the expression of the essential cell cycle regulators Cdc25c and Cyclin B1 [140]. 

#### 3.1.3. Tumor Suppression and Proliferation Inhibition

Conversely, some lncRNAs act as tumor suppressors and are often downregulated in OC cells. Their decreased expression contributes to the malignancy and progression of OC. The lncRNA GAS5, for instance, functions as a tumor suppressor and serves as a prognostic marker in OC [176]. It binds to heterogeneous nuclear ribonucleoprotein K (hnRNPK), stabilizing it and inhibiting the downstream p-AKT pathway, thereby reducing OC cell proliferation [55]. Another tumor-suppressor lncRNA, MEG3, sequesters miR-376a, which promotes cell proliferation by targeting Krüppel-like factor 15 (KLF15) and Caspase-8 [177]. The reduced expression of MEG3 in OC patient samples correlates with higher levels of miR-376a and increased cell proliferation [178]. Similarly, tumor-suppressor lncRNAs such as LINC-PINT, TUSC7, and ADAMTS9-AS2 downregulate the proliferation of OC cells through different mechanisms by modulating genes that regulate the cell cycle and proliferation [154,161,168].

### 3.2. LncRNAs in Ovarian Cancer Cell Survival

LncRNAs promote tumor cell survival through various mechanisms. Cancer cells adapt to hostile environments by evading or suppressing programmed cell death (apoptosis) and autophagy [179]. Aberrant regulation of signaling pathways such as mTOR, KRAS, and MAPK promotes cell survival and proliferation, the hallmarks of cancer cells [180]. In OC, lncRNAs alter gene expression to create a tumor microenvironment (TME) conducive to growth and survival.

#### 3.2.1. Evasion of Apoptosis

LncRNAs can promote OC cell survival by inhibiting apoptosis, thus allowing cancer cells to evade programmed cell death. The lncRNA LINC00152 is upregulated in the HO-8910, A2780, and SVOV3 OC cell lines, where it enhances cell survival by stimulating the increased expression of myeloid cell leukemia (MCL-1), an anti-apoptotic protein. LINC00152 achieves this by sequestering miR-125b, which normally targets MCL-1. By preventing cytochrome C release from mitochondria, MCL-1 attenuates the activity of the apoptotic protein family Bcl-2, thereby restricting apoptosis [112]. The highly upregulated lncRNA HULC increases tumorigenesis by decreasing the apoptotic rate in the OVCAR3 and A2780 OC cell lines. HULC interacts with ATG7 to induce a reduction in the expression of the autophagy-related components LC3-II and LAMP1, reducing apoptosis and enhancing cell survival [181].

Although only a few lncRNAs are known to induce apoptosis in OC, MEG3 exhibits a pro-apoptotic role in OC cells. As a tumor-suppressor lncRNA, MEG3 acts as a ceRNA against miR-205-5p, inhibiting cell proliferation by downregulating the expression of PTEN and SMAD4 in the SKOV3 and OVCAR8 OC cell lines [182].

#### 3.2.2. Regulation of Ferroptosis

Ferroptosis is a distinct form of programmed cell death characterized by iron-dependent lipid peroxidation, and some lncRNAs play a key role in inhibiting this process, thereby supporting tumor cell survival. The lncRNA CACNA1G-AS1 has been identified as a key player in inhibiting ferroptosis, which supports the survival and growth of tumor cells. CACNA1G-AS1 achieves this by upregulating ferritin heavy chain 1 (FTH1), which in turn leads to a reduction in mitochondrial autophagy. This effect is further amplified by the increased expression of IGF2 mRNA Binding Protein 1 (IGF2BP1). Together, these actions enhance tumor cell survival in OC cell lines, specifically SKOV3 and A2780, providing evidence for its role in cancer cell survival and cancer progression [183].

#### 3.2.3. Modulation of Autophagy

Autophagy serves dual roles in cancer, both suppressing and supporting tumor survival under stress conditions such as nutrient deprivation, hypoxia, and chemotherapy [184]. LncRNAs modulate autophagy in OC, either promoting or inhibiting this process to influence cell survival. The lncRNA RNF157-AS1 acts as an oncogene by inhibiting autophagy in SKOV3 cells to promote cell survival. It functions as a molecular scaffold, tethering chromatin modifiers like Enhancer of Zeste Homolog 2 (EZH2) and High mobility AT-Hook 1 (HMGA1), which suppresses the mTOR pathway and key autophagy proteins such as Unc-51-like Autophagy Activating Kinase 1 (ULK1) and DIRAS Family GTPase 3 (DIRAS3) [185].

Conversely, overexpression of the tumor-suppressor lncRNA GAS8-AS1 has been found to induce autophagy in the SKOV3 and A2780 OC cell lines by increasing the level of LC3II expression. In addition, the binding of GAS8-AS1 with Beclin1, an essential component for the formation of autophagosomes, increases the apoptotic rate in OC [186].

### 3.3. LncRNAs in Ovarian Cancer Metabolic Reprogramming

Metabolic reprogramming refers to the alteration of cellular metabolism to meet the demands of cancer cells [187]. In cancer cells, the metabolic pathways are reprogrammed to augment the biosynthesis of macromolecules, enhance cellular ATP/energy levels, and provide redox homeostasis within the cells to promote tumor genesis and progression, and lncRNAs play an indispensable role in regulating these functions [18,188]. In OC cells, lncRNAs play a cardinal role in regulating major cellular metabolic pathways such as those of glucose, lipids, and amino acids to promote cancer progression.

#### 3.3.1. Regulation of Glucose Metabolism

Even under normoxic conditions, OC cells favor lactate production from glucose, promoting glycolysis and altering OXPHOS (oxidative phosphorylation) to augment the energy supply to facilitate tumor cell proliferation, metastasis, and therapy resistance [189]. LncRNAs play a key role in this process. In OC cells, the lncRNA SNHG3 sequesters miR-186-5p, leading to the upregulation of the translation factor EIF4AIII and its downstream targets, such as PKM, PDHB, IDH2, and UQCRH. These targets enhance the tricarboxylic acid (TCA) cycle and OXPHOS, thereby meeting the energy demands of OC cells [190]. Similarly, the lncRNA NRCP has been shown to upregulate the expression of the glycolytic enzyme, glucose-6-phosphate isomerase (GPI), by promoting the interactions between STAT1 and RNA Pol II in the *GPI* gene promoter to promote its transcription and tumor progression [191].

#### 3.3.2. Regulation of Fatty Acid Metabolism

OC cells often prefer fatty acid metabolism for energy needs over glucose metabolism and hence metastasize to the lipid-enriched omental fat pad [192]. Despite the recognized importance of fatty acid metabolism in OC, research on lncRNAs regulating these mechanisms in OC is still emerging. Recent studies involving bioinformatics predictive models and computational analysis based on the TCGA patient data have identified a candidate lncRNA, LINC00861, that regulates fatty acid metabolism in OC and can serve as a prognostic signature. However, detailed mechanistic studies are needed to understand its precise mechanisms in regulating fatty acid metabolism driving OC progression [193,194].

#### 3.3.3. Regulation of Amino Acid Metabolism

LncRNAs also play a critical role in regulating amino acid metabolism to promote OC progression. For instance, circ_0025033 sequesters miR-370-3p, leading to the upregulation of the sodium-dependent neutral amino acid transporter SLC1A5, which enhances amino acid metabolism [195]. In contrast, the tumor-suppressor lncRNA SLC7A11-AS1, which is downregulated in OC, normally acts as antisense regulator of *SLC7A11* gene [155]. SLC7A11 encodes a sodium-independent anionic amino acid transporter that, when upregulated in cancer cells, promotes glutathione production, regulates glutamine metabolism, and maintains redox homeostasis, all of which contribute to OC progression [155]. 

#### 3.3.4. Intercellular Metabolic Signaling in the Tumor Microenvironment

In addition to reprogramming cancer cell metabolism, lncRNAs are involved in intercellular signaling within the TME, influencing both cancer cells and stromal components such as cancer-associated fibroblasts (CAFs). In OC, CAFs within the pro-metastatic niche synthesize and release CXCL14, which stimulates OC cells through paracrine signaling to upregulate LINC00092. LINC00092 binds to the PFKFB2 protein, stabilizing and upregulating PFKFB2 expression and promoting glycolysis in OC cells. This metabolic shift triggers the release of secretory signals from OC cells that, in turn, regulate CAFs, maintaining CAF-specific markers such as αSMA and FAP, thereby promoting OC metastasis [196]. LncRNAs also play a role in the metabolic reprogramming of CAFs within the TME. For example, small extracellular vesicles (sEVs) containing UCA1, released from OC cells, are taken up by stromal fibroblasts, leading to enhanced glucose metabolism in peri-tumoral fibroblasts through the upregulation of HK2 and GLUT2 [197]. This intercellular signaling highlights how lncRNAs within sEVs contribute to tumor progression through metabolic reprogramming.

### 3.4. LncRNAs in Ovarian Cancer Cell Migration

Cellular migration is critical for the metastatic spread of OC, allowing cancer cells to spread from the primary site to distant locations in the body. This process involves the degradation of the extracellular matrix proteins like integrins, facilitating cellular migration and disrupting cellular adhesion. EMT is a key development in this process, where epithelial cells lose their polarity and adhesion properties and acquire mesenchymal characteristics, making them more mobile and invasive. This facilitates the escape of cancer cells from the primary tumor site into the blood vessels, disseminating to distant organs and forming metastases [198].

#### 3.4.1. Promotion of EMT by Oncogenic LncRNAs

EMT is a fundamental process in cancer metastasis, and several lncRNAs play crucial roles in promoting this transition in OC [199]. The lncRNA SNHG1 is overexpressed in A2780 and SKOV3 OC cells, where it promotes EMT by sequestering miR-454, leading to the upregulation of ZEB1. ZEB1 is a key transcription factor that suppresses E-cadherin, an epithelial cell marker, while promoting mesenchymal characteristics, as evidenced by the increased expression of mesenchymal markers such as N-cadherin, vimentin, MMP-2, and MMP-9 [137]. In a recent study, the lncRNA AC005224.4 was shown to be highly upregulated and associated with EMT and metastasis in the SKOV3 and CaOV3 OC cell lines. AC005224.4 sequesters miR-140-3p, allowing increased expression of the transcription factor SNAI2 which results in the increased expression of several EMT markers: vimentin, Snail, And N-cadherin [200]. HOTAIR, a widely studied lncRNA in OC, plays multiple roles in OC progression including EMT [201]. In vitro and in vivo studies have shown that HOTAIR promotes pro-metastatic effects in OC through the regulation of EMT-associated genes and MMPs [202].

#### 3.4.2. Inhibition of EMT by Tumor-Suppressor LncRNAs

Tumor-suppressor lncRNAs often counteract EMT, thereby inhibiting cancer cell metastasis. LINC-PINT is a lncRNA that is downregulated in OC and acts as a ceRNA for miR-374a-5p. By sequestering miR-374a-5p, LINC-PINT upregulates FOXO1, a transcription factor that inhibits vimentin (a mesenchymal marker) and promotes E-cadherin expression (an epithelial marker), thereby suppressing EMT in OVCAR3 and A2780 cells [161,203]. LIFR-AS1 is another tumor-suppressor lncRNA that inhibits EMT by downregulating Snail and N-cadherin, both of which are critical for mesenchymal transition, while upregulating E-cadherin in A2780 and SKOV3 cells [160].

#### 3.4.3. Direct Regulation of Cell Migration

In addition to regulating EMT, lncRNAs directly influence the migration of OC cells, which is essential for metastasis. Several lncRNAs not only modulate the expression of the components of different signaling pathways such as Wnt/β-catenin, PI3K/AKT, and TGF-β but also upregulate the expression of different cytoskeletal regulators and matrix-remodeling enzymes involved in cell migration and invasion [199,204]. The lncRNA HOTAIR not only promotes EMT but also enhances cell migration by sequestering miR-206. This sequestration leads to increased expression of Cyclin D1 (CCND1) and Cyclin D2 (CCND2), which are involved in cell migration and invasion along with their role in cell proliferation in the SKOV3, OVCAR3, COV362, and A2780 OC cell lines [205].

Conversely, tumor-suppressor lncRNAs that inhibit cellular migration are often downregulated in OC. LINC-PINT, an lncRNA expressed at low levels in OC, acts as a ceRNA for miR-374a-5p which downregulates its target, the Foxhead box O1 (*FOXO1*) gene, which regulates cell migration by inhibiting vimentin and promoting E-cadherin expression in OVCAR3 and A2780 cells [161,203]. Similarly, the lncRNA TUSC7 inhibits cellular migration in SKOV3 cells by sequestering miR-616-5p, which negatively regulates Glycogen Synthase Kinase 3 beta (GSK3β), leading to the activation of the β-catenin pathway and promoting OC cell migration [168]. The tumor-suppressor lncRNA MEG3 positively regulates PTEN and reduces cell migration in SKOV3 OC cells through the induction of cell apoptosis [206].

### 3.5. LncRNAs in Ovarian Cancer Angiogenesis

Angiogenesis, the formation of new blood vessels, is a crucial process by which cancer cells meet their increasing demands for nutrients and oxygen, facilitating their growth and progression. This process is tightly regulated by a balance of pro- and anti-angiogenic factors [207]. Aberrant expression of lncRNAs can disrupt this balance and promote tumor angiogenesis in the TME [208].

#### 3.5.1. Promotion of Angiogenesis by Oncogenic LncRNAs

Oncogenic lncRNAs can enhance angiogenesis by modulating the expression of pro-angiogenic factors. For example, the lncRNA DANCR promotes tumor angiogenesis by sequestering miR-145, which normally targets VEGF. By inhibiting miR-145, DANCR upregulates VEGF expression in the SKOV3 and A2780 OC cell lines, contributing to increased tumor angiogenesis [209]. Another example is the sEV-packaged lncRNA ATB, which is released by the OC cell lines SKOV3 and A2780. This lncRNA promotes angiogenesis in human umbilical vein endothelial cells (HUVECs) by sequestering miR-204-3p. This sequestration prevents miR-204-3p from downregulating Transforming Growth Factor β Receptor 2 (TGFβR2), thereby altering the TME in HUVECs and enhancing angiogenesis [82]. The lncRNA NEAT1 is associated with the upregulation of several angiogenesis-related gene expressions, including *Sema4D*, *Tiam1*, *Rac1*, and *Plexin B1*, in the SKOV3 and A2780 OC cell lines. Another study on NEAT1 has shown that it specifically promotes angiogenesis by sequestering miR-214-3p, which results in the increased expression of its target, SEMA4D, thereby enhancing malignancy and angiogenesis in OC [210]. The lncRNA TUG1 promotes angiogenesis in the SKOV3 and CaOV3 OC cell lines by the positive regulation of leucine-rich α-2-glycoprotein-1 (LRG1). LRG1 binds to the TGF-β receptor, additionally activating certain angiogenic factors, for example, VEGFA and Ang-1 [146].

#### 3.5.2. Inhibition of Angiogenesis by Tumor-Suppressor LncRNAs

In contrast, tumor-suppressor lncRNAs inhibit tumor angiogenesis. One such lncRNA is MEG3. It has been demonstrated that the overexpression of MEG3 in ovarian carcinoma-derived microvascular endothelial cells (ODMECs) resulted in the suppression of angiogenesis. MEG3 competitively binds with miR-376a, preventing it from targeting YBX1 and allowing the expression of RAS p21 protein activator 1 (RASA1), a known suppressor of angiogenesis [178]. This mechanism highlights the role of MEG3 in maintaining angiogenic balance within the TME and preventing tumor angiogenesis.

### 3.6. LncRNAs in Ovarian Cancer Stemness

Cancer stem cells (CSCs) within OC represent a subpopulation of OC cells with unique properties that enable them to withstand harsh environmental conditions such as hypoxia within the TME. CSCs can evade therapy by altering different signaling pathways including Wnt, Notch, KRAS, and PTEN, among others [211]. Several CSC markers have been identified in OC, including CD133, CD117, ALDH, EpCAM, SOX2, and Nanog Homeobox (NANOG), all of which play a critical role in the initiation and progression of the disease. These markers play a critical role in the initiation and progression of the disease and are used to identify and isolate the CSCs responsible for tumor initiation, therapy resistance, EMT, and metastasis [212]. Several lncRNAs have been studied for their role in conferring stemness in OC cells. These lncRNAs function through several molecular mechanisms, including the epigenetic modulation of different signaling pathways, sequestration of several tumor-suppressor miRNAs, and transcriptional regulation of different oncogenes [213].

#### 3.6.1. Transcriptional Regulation of Stemness by LncRNAs

LncRNAs play a crucial role in regulating the transcription of genes that confer stemness in OC cells. For instance, the lncRNA HOTAIR positively regulates the expression of transcription factor T-box transcription factor 3 (TBX3), which in turn transactivates the expression of SRY-box 2 (SOX2) and Octamer-binding transcription factor 4 (Oct4). These factors are essential for maintaining pluripotency and self-renewal in the CSC population in the SKOV3, ES2, and OVCAR3 OC cell lines [214,215]. This regulation promotes EMT and sustains the stem-like properties of CSCs, which are key drivers of tumor progression and therapy resistance.

#### 3.6.2. Epigenetic Modulation of Stemness by LncRNAs

Epigenetic regulation by lncRNAs is another mechanism through which CSCs maintain their stemness. MALAT1 is an lncRNA that functions as both a transcriptional and epigenetic regulator, effectively stabilizing YAP protein expression to promote stemness in the cisplatin-resistant SKOV3 OC cell line. The overexpression of YAP can reverse the effects of MALAT1 knockdown, leading to increased expression of stemness markers such as aldehyde dehydrogenase 1 (ALDH1) and NANOG. However, the exact mechanisms through which YAP regulates stemness in OC remain to be fully defined [216].

#### 3.6.3. Regulation of CSC Signaling Pathways by LncRNAs

LncRNAs modulate key signaling pathways to enhance CSC properties and promote stemness in OC. LINC00115, for example, upregulates the expression of SOX9 by sequestering miR-30a-5p. This interaction results in an increased CSC population and decreased apoptosis in A2780 and CD133+ OC cells via the Wnt signaling pathway. The suppression of LINC00115 leads to decreased levels of CSC markers, including CD44, NANOG, and CD133, as well as SOX9, highlighting its role in promoting CSC-related traits and therapy resistance [217].

### 3.7. LncRNAs in Ovarian Cancer Associated with Immune Evasion

Immune evasion is a critical mechanism that allows cancer cells to avoid detection and destruction by the body’s immune system. This process can occur early in tumor development, with cancer cells adopting various strategies to evade immune surveillance. The human immune system consists of two main components: innate and adaptive immune responses. The innate immune response provides the initial defense against pathogens and includes physical barriers (such as skin, mucous membranes, and gastric juices) and immune cells such as macrophages, natural killer cells, and neutrophils [218]. In contrast, the adaptive immune response is highly specific and involves T lymphocytes (T cells) and B lymphocytes (B cells) [123]. LncRNAs play a significant role in manipulating these immune responses, thereby facilitating immune evasion in OC.

#### 3.7.1. Modulation of Innate Immunity

LncRNAs can modulate the innate immune response, enabling OC cells to evade initial immune detection. A prime example is the lncRNA IL21-AS1, which is highly upregulated in both OC patient tumor samples and SKOV3 and A2780 OC cells. IL21-AS1 promotes immune evasion by upregulating CD24 expression through the sequestration of miR-561-5p. CD24 inhibits macrophage-mediated phagocytosis by binding to the Siglec-10 receptor on macrophages, thus preventing the immune system from effectively targeting the OC cells [219]. Another example involves the lncRNA MEG8, which has been implicated in the downregulation of sine oculis-binding protein (SOBP) in OC. Bioinformatics analyses of OC patient databases (GSE36668, GSE12470, GSE14407, and GSE27651) suggest that MEG8 could sequester miR-378, leading to the upregulation of SOBP and associated cytokines, which are involved in immune pathways. This interaction highlights a potential role for MEG8 in facilitating immune evasion in OC cells [220].

#### 3.7.2. Modulation of Adaptive Immunity

LncRNAs also play a role in modulating adaptive immunity, allowing OC cells to evade more specific and targeted immune responses. One such lncRNA is SNHG12, which has been shown to suppress T-cell activity in OC. In patient samples, SNHG12 was found to induce the overexpression of IL-6-mediated PD-L1 expression in cancer cells. SNHG12 recruits the transcription factor NF-κB1 to the *IL-6* gene promoter region, enhancing IL-6 expression and subsequently increasing PD-L1 levels, which suppresses T-cell function and contributes to immune evasion [221].

### 3.8. LncRNAs in Ovarian Cancer Therapy Resistance

Therapy resistance in OC is a multifaceted and complex phenomenon. LncRNAs contribute significantly to this process by modulating gene expression related to drug uptake, efflux, metabolism, and resistance to radiotherapy, thereby reducing the effectiveness of therapeutic interventions [222].

#### 3.8.1. Modulation of Drug Efflux and Uptake

LncRNAs play a crucial role in regulating the expression of genes involved in drug efflux and uptake, leading to reduced intracellular concentrations of therapeutic drugs. For example, LINC01118 is highly expressed in OC patient tissue samples and upregulates ATP-Binding Cassette C1 (ABCC1) drug efflux transporters by sequestering miR-134, a negative regulator of ABCC1. This leads to paclitaxel resistance in OC [223]. Similarly, the lncRNA WDFY3-AS2 is overexpressed in cisplatin-resistant A2780 OC cells and sequesters miR-139-5p, which modulates the expression of Syndecan-4 (SDC4), a transmembrane heparan sulfate proteoglycan that upregulates drug efflux pumps. This contributes to cisplatin resistance in OC cells [224].

#### 3.8.2. Alteration of Drug Metabolism

Several other lncRNAs are involved in conferring therapy resistance by altering drug metabolism. The knockdown of the lncRNA PVT1 lowered the levels of aldo-keto reductase (AKR) family 1 member C1 (AKR1C1), AKR1C2, and AKR1B10 in the SKOV3 OC cell line, which plays a role in breaking down therapeutic agents such as paclitaxel, cisplatin, and doxorubicin into simpler non-toxic compounds [225]. Transcriptionally, the downregulation of PVT1 has been associated with doxorubicin sensitivity in the SKOV3 OC cell line, although the regulation of AKR enzymes by the lncRNA PVT1 is yet to be studied [135].

The lncRNA H19 promotes cisplatin resistance through the activation of the glutathione synthesis pathway (GSH) in A2780 and A2780 cisplatin-resistant OC cells. It stabilizes Nrf2, a key factor in the activation of Glutathione S-transferase Pi 1 (GSTP1), which positively regulates the GSH pathway, leading to cisplatin inactivation [98]. It has also been shown that UCA1 acts as a ceRNA for miR-143, resulting in the upregulation of FOS Like 2, AP-1 Transcription Factor Subunit (FOSL2) in cisplatin-resistant A2780 and SKOV3 OC cell lines. However, the mechanism through which FOSL2 regulates cisplatin resistance in OC is yet to be studied [152].

#### 3.8.3. Role of Tumor-Suppressor LncRNAs in Therapy Sensitivity

In contrast to these resistance-inducing lncRNAs, the expression of a few tumor-suppressor lncRNAs can be correlated with therapy sensitivity in OC. For example, curcumin-treated OC cells showed overexpression of the tumor-suppressor lncRNA MEG3, both in OVCAR3 and SKOV3 cells as well as the sEVs derived from them. MEG3 sequesters and reduces the levels of miR-214 both in the cell lines and in the sEVs, which is known to confer chemoresistance in OC cells through the activation of the Akt pathway through its interaction with PTEN. The overexpression of MEG3 has been shown to enhance chemosensitivity in the cisplatin-resistant A2780 cell line [226]. LINC00312 is downregulated in SKOV3 cisplatin-resistant cell lines when compared to SKOV3 cells. Its overexpression induced sensitivity to the cisplatin-resistant SKOV3 cells through the activation of the caspase-3 apoptotic pathway and downregulated expression of MDR1 membrane proteins [227].

In another instance, LINC01508 has been shown to sensitize OC cells to cisplatin by modulating the AKT pathway and downregulating the YAP–Hippo pathway. Studies with the syngeneic sensitive and resistant cell OV2008 cell lines have indicated that the overexpression of LINC01508 sensitizes the OC cells to cisplatin by inhibiting the Yap–Hippo signaling pathway. In these cells, LINC01508 inhibits YAP signaling through an as-yet undefined signaling mechanism [228]. It has also been shown that LINC01125, expressed at a lower level in cisplatin-resistant SKOV3 and A2780 OC cell lines, confers cisplatin sensitivity when overexpressed. The overexpression of LINC01125 sequesters miR-1972, a highly expressed miRNA, in both OC patient samples and cell lines. Although the oncogenic properties of miR-1972 are yet to be studied, through the use of the TargetScan tool, it has been deduced that miR-1972 is involved in the regulation of targets involved in the apoptotic pathway [229].

#### 3.8.4. LncRNAs and Radiotherapy Resistance

Although radiotherapy is not commonly used in OC, in vitro experiments have shown that lncRNAs can contribute to radiotherapy resistance. Radiotherapy resistance primarily involves the ability of cancer cells to enhance their DNA repair abilities via proteins such as ATM and BRCA1/2 [230]. In addition, cancer cells can evade radiotherapy-induced apoptosis by upregulating anti-apoptotic proteins such as BCL-2 [231]. The hypoxic TME further contributes to resistance by stabilizing hypoxia-inducible factors that promote cell survival [222]. Few lncRNAs have been studied for their role in conferring radiotherapy resistance in OC cells. Specifically, the lncRNA FAM83H-AS1 has been shown to interact with Human Antigen R (HuR), an RNA-binding protein, stabilizing it and thus contributing to radio-resistance in the SKOV3, A2780, and SW626 OC cell lines. The knockdown of FAM83H-AS1 restores radiosensitivity in these cells [232].

## 4. Clinical Implications

LncRNAs are gaining greater importance owing to their pivotal roles in OC, functioning either as oncogenes or tumor suppressors. Their clinical relevance extends from early detection to specialized therapeutic strategies, highlighting the transformative impact of lncRNA research in clinical settings. The differential expression of lncRNAs in cancer cells and tissues presents opportunities for early cancer detection, prognosis, and treatment optimization. Specific lncRNAs associated with aggressive tumor behavior can provide insights into optimizing therapeutic approaches [233,234]. The exploitation of lncRNA expression profiles for patient stratification represents a promising frontier in personalized medicine for OC. Identifying lncRNA signatures associated with specific subtypes, disease stages, or treatment responses could lead to tailored therapeutic approaches, ultimately enhancing therapeutic efficacy and patient outcomes.

### 4.1. LncRNAs as Diagnostic Biomarkers in Ovarian Cancer

The differential expression of lncRNAs in cancer cells compared to normal cells makes them potential biomarkers for OC. For example, the lncRNA LOXL1-AS1 shows heightened expression in EOC patients and is linked to poor patient survival, especially in advanced cancer stages [235]. In addition, the overexpression of the lncRNA XIST in 98 EOC patient tissue samples supports its role as an important diagnostic biomarker in OC. Further, the pro-tumorigenic role of XIST was validated in the OV90, OVCAR3, SKOV3, and A2780 OC cell lines [236]. In another study, GSEA data from the GSE10971, GSE29450, and GSE54388 datasets were analyzed and a panel of four lncRNAs, LINC00511, LINC01132, RP11-83A24.2, and MIR762AG, were found to be highly expressed in OC patients and were correlated with low survival by Cox regression analysis. Further intensive study of LINC00511 revealed that it interacts with EZH2 and mediates the H3K27me3 modification of the promoter of *p21*, an essential regulator of CDKN1A [237]. The exploration of lncRNAs as diagnostic biomarkers is ongoing, with continuous research unraveling new biomarkers and their roles in disease progression. The use of lncRNAs as biomarkers holds great promise for early detection and personalized therapy in OC.

### 4.2. LncRNAs as Prognostic Indicators and Predictors of Treatment Response

Aberrant expression of lncRNAs can serve as a prognostic indicator for patient survival, disease progression, and tumor recurrence. Studies utilizing the TCGA database have indicated that the increased expression of LOC101927151 in OC patients is associated with a poor prognosis, while the decreased expression of LINC00861 and LEMD1-AS1 correlates with unfavorable outcomes [238]. A similar TCGA database-based analysis has identified that the increased expression of the lncRNAs RP4-700P18.3, RP11-57P19.1, RP11-307C12.11, RP11-254I22.1, RP1-223E5.4, and GACAT3 is associated with better tumor survival, while a higher expression of the lncRNAs PTPRD-AS1 and RP11-80H5.7 is linked with poor survival in OC patients [239]. OC progression and recurrence are also tied to aberrant lncRNA expression. The novel lncRNA LINC00565 is highly expressed in OC, and its knockdown inhibited the metastatic nature of suggesting a strong prognostic potential for this lncRNA [240]. A LASSO regression analysis of the GSE9891 and GSE30161 GSEA datasets indicated that a panel of six lncRNAs, namely RUNX1-IT1, MALAT1, H19, HOTAIRM1, LOC100190986, and AL132709.8, were associated with OC recurrence. All of these lncRNAs exhibited a heightened expression in late-stage OC patients and were shown to be associated with a higher risk of recurrence [241].

### 4.3. LncRNAs as Therapeutic Targets

A study involving 266 OC patients revealed that overexpression of the lncRNAs LINC00472, ASP1-IT1, and FAM215A is associated with early-stage disease and low-grade tumors. These lncRNAs were found to play a role in inhibiting cellular proliferation offering insights into potential therapeutic targets [242]. In another study, the differentially expressed lncRNAs in EOC patient data from 22 cohorts including GEO datasets and GEPIA2 were analyzed. A total of eight noteworthy novel upregulated lncRNAs were discovered that can serve as potential diagnostic targets, including ENSG00000187951, ENSG00000285756, LINC01297, LINC01770, LINC01977, MIR205HG, TFAP2A-AS1, and ZNF232-AS1. Additionally, the lncRNAs MIR924HG and GUSBP11 were associated with an unfavorable prognosis [243].

### 4.4. Therapeutic Potential of Targeting lncRNAs in Ovarian Cancer

Targeting lncRNAs in OC holds significant promise as a novel therapeutic strategy. Unlike conventional therapies that focus on single signaling nodes, targeting lncRNAs offers the potential to disrupt complex networks of pathways involved in cancer genesis and progression. LncRNAs modulate multiple cellular processes, including chemoresistance, autophagy, EMT, DNA damage repair, and apoptosis, making them particularly attractive as therapeutic targets. This multifunctional role could lead to more effective and multi-targeted therapeutic strategies [233,244].

One key advantage of targeting lncRNAs is their ability to adapt and perform different functions under varying conditions, such as hypoxia or inflammation within the TME. This adaptability allows for a more dynamic and robust therapeutic approach [245]. Emerging strategies for targeting lncRNAs in OC include the use of antisense oligonucleotides (ASOs), LNAgapmers, RNA interference (RNAi) technologies, and small-molecule inhibitors. These approaches aim to modulate lncRNA function by either inhibiting their expression or blocking their interactions with other biomolecules [246].

ASOs are short, synthetically designed nucleotide sequences that bind to specific lncRNAs, leading to their degradation or inhibition. LNAgapmers, which are ASOs modified with locked nucleic acids, provide greater stability and binding affinity, enhancing their ability to silence lncRNAs. RNAi technologies, including siRNA and shRNA, utilize small RNA molecules to interfere with specific lncRNAs, reducing their expression and altering cancer cell function. Small-molecule inhibitors, on the other hand, disrupt lncRNA activity by interfering with their interactions with other proteins and signaling pathways [247].

Clinical trials involving RNA-targeting therapeutics have shown the feasibility of these strategies. For example, ASOs targeting survivin in prostate cancer [248] and c-Raf kinase in recurrent OC [249] demonstrate the potential for these approaches to be applied to lncRNA-targeted therapies in OC. Similarly, siRNAs targeting several protein and tyrosine kinases in cancer are actively being tested in clinical trials. Notably, liposome-encapsulated EphA2-targeting siRNA is currently in Phase I clinical trials for solid tumors, including ovarian cancer (NCT01591356) [250]. Although lncRNAs have been explored for their high translational potential, both in vitro and in vivo, further extensive studies have to be carried out to bring them to the stage of clinical trials.

## 5. Summary and Perspectives

This review highlights the growing importance of lncRNAs as promising biomarkers and therapeutic targets in OC. Extensive research has provided valuable insights and revealed exciting opportunities for further exploration. Moving forward, validating lncRNA biomarkers across diverse patient cohorts and clinical settings is crucial. The variability in study designs, sample sizes, and analytical methods underscores the need for standardized protocols and rigorous validation frameworks to ensure the reliability and reproducibility of lncRNA biomarkers.

Most of the mechanistic studies on lncRNA are carried out, in vitro and in vivo, by either knocking down or overexpressing the lncRNAs and analyzing their functional roles. While these approaches provide valuable insights, they often fail to account for the endogenous context of lncRNA expression and the potential compensatory mechanisms that may occur. More recently, studies have utilized knockout (KO) techniques, such as CRISPR, to eliminate specific lncRNAs and perform functional assays. For example, CRISPR-mediated UCA1 KO mouse models were shown to develop significantly smaller tumors compared to wild-type mice For instance, UCA1 KO mice models using CRISPR were demonstrated to develop significantly smaller tumors when compared with the wild-type mice [66]. In another study, a HOTAIR KO cell line showed increased sensitivity towards cisplatin and a reduction in the OC stem cell population [251]. Similarly, a knockout of the lncRNA HOXA11-AS in cisplatin-resistant A2780 OC cells showed increased autophagy and cisplatin sensitivity [103]. These emerging technologies underscore the potential of lncRNA knockout models to provide deeper insights into their functional roles in OC progression.

**Figure 6:**
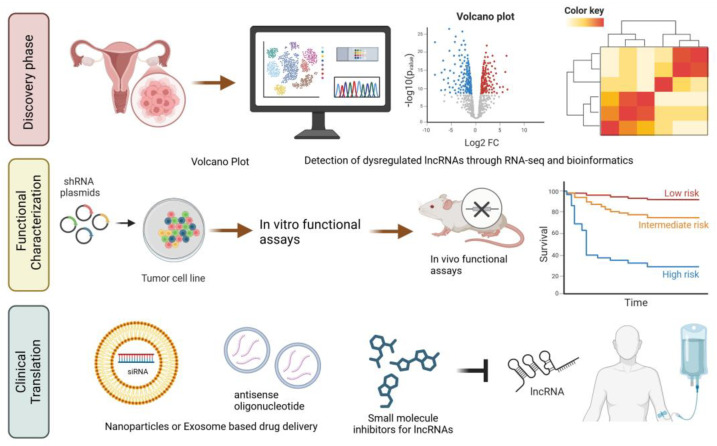
Roadmap of lncRNA research in ovarian cancer. The roadmap can be divided in three main phases: The discovery phase involves identifying dysregulated lncRNAs through RNA sequencing (RNA-seq) and bioinformatics tools, as shown by volcano plots and clustering analyses. The functional characterization phase includes validating lncRNAs as biomarkers and elucidating their roles through in vitro functional assays using tumor cell lines and in vivo studies in animal models, linking lncRNA expression to patient survival. Finally, the clinical translation phase highlights therapeutic strategies, such as nanoparticle- or exosome-based delivery of antisense oligonucleotides (ASOs) and siRNAs, and the development of small-molecule inhibitors targeting specific lncRNAs for clinical applications.

The complex roles and intricate molecular mechanisms of lncRNAs in OC pathogenesis offer rich opportunities for discovering novel diagnostic, prognostic, and therapeutic targets. Emerging technologies, such as advanced bioinformatics and single-cell sequencing, present unprecedented opportunities to identify these targets. The integration of lncRNA studies with other omics data (genomics, proteomics, or metabolomics) could provide a more integrated view of the molecular landscape of OC. This network-based approach could uncover novel lncRNA-associated pathways and interactions, offering deeper insights into OC pathobiology and informing more effective treatment strategies (Figure 6).

Translating in vitro findings to in vivo systems remains a significant challenge in lncRNA research due to the low sequence conservation between human and mouse lncRNAs. This lack of conservation often results in differences in expression, structure, and function, limiting the utility of mouse models and causing potential off-target effects with therapeutics like ASOs or siRNAs. To address these challenges, several strategies can be employed.

Parallel studies in mouse cells can help to evaluate functional equivalents of human lncRNAs. Humanized mouse models, engineered to express human lncRNAs in their native regulatory context, can provide more accurate in vivo data. Human organoid models also present a promising alternative, enabling tissue-specific analyses of lncRNA functions while reducing dependency on animal models. Human organoid models also offer a promising alternative, allowing tissue-specific analysis of lncRNA functions while reducing dependency on animal models.

Organoids can also be used in co-culture systems to study lncRNAs’ roles in the tumor microenvironment. CRISPR/Cas-based gene-editing technologies offer another solution by enabling the precise replacement of mouse lncRNAs with their human counterparts in transgenic models. Furthermore, computational tools can identify conserved functional domains or motifs, guiding the design of cross-species therapeutics with minimal off-target effects. By integrating humanized models, organoids, advanced gene-editing, and bioinformatics, the conservation barrier can be overcome, enabling the accurate characterization of human lncRNAs and advancing their clinical translation.

Machine learning (ML) and artificial intelligence (AI) are poised to play transformative roles in the therapeutic targeting and clinical utility of lncRNAs. By analyzing large datasets, including multi-omics data, ML algorithms can identify patterns and predict lncRNA functions, interactions, and their roles in therapy resistance or disease progression. AI-driven models can also aid in the discovery of novel lncRNAs and predict their potential as therapeutic targets. Furthermore, AI can optimize personalized medicine by integrating lncRNA profiles with clinical data to predict patient responses to specific treatments, thereby enhancing treatment efficacy and minimizing adverse effects.

As we move forward, these advancements are anticipated to open new avenues for the development of lncRNA-based diagnostics, prognostics, and therapeutic interventions. It can be envisioned that lncRNA-based personalized medicine will become a cornerstone in the fight against OC, offering tailored precision cancer therapies and significantly improving patient outcomes and quality of life.

## Figures and Tables

**Table 1:** LncRNAs that act as oncogenes in ovarian cancer.

	LncRNA	Position/Chromosomal Location	Function	Mechanism
1.	ADAMTS9-AS1	Antisense *chr3p14.1 (64,547,014-64,573,878)*	Inhibits ferroptosis resulting in increased cell proliferation and migration	Acts as a ceRNA to miR-587, downregulating the expression of SLC7A11 [80]
2.	ANRIL	Antisense *chr9p21.3 (21,994,777-22,121,096)*	Promotes cell proliferation and invasion.	Activation of the Wnt/β-catenin pathway [81]
3.	ATB	Sense *chr14q11.2 (19,126,530-19,128,974)*	Promotes tumorigenesis	Acts as a ceRNA towards miR-204-3p, upregulating the TGFβR2 pathway [82]
4.	AWPPH	Intergenic * chr2q13 (111,005,119-111,659,454) *	Promotes overall OC development	Upregulates β-catenin expression by activating the Wnt/β-catenin pathway [83]
5.	CCAT1	Antisense *chr8q24.21 (127,207,381-127,219,268)*	Confers cisplatin resistance	Acts as a ceRNA against miR-454, inducing the expression of survivin [84]
			Induces EMT of OC cells	Upregulates TGFβR1 through the sponging of miR-490-3p [85]
			Contributes to metastasis and progression in epithelial ovarian cancer (EOC)	Modulates the ADAM17/Wnt1/STAT3/ZEB1 regulatory network via miR-152 and miR-130b [86]
			Promotes proliferation of OC	Sequesters miR-1290 and suppresses its tumorigenic role [87]
6.	CCAT2	Intergenic *chr8q24.21 (127,400,398-127,402,150)*	Acts as an oncogene	Sequesters miR-424, resulting in its downregulation [88]
7.	CCEPR	Bidirectional *chr10q21.1 (59,173,190-59,176,464)*	Promotes cellular invasion and poor prognosis	Activation of the Wnt/β-catenin pathway [89]
8.	CHRF	Intergenic *chr15q13.2 (30178876-30179943)*	Confers cisplatin resistance	Acts as ceRNA to miR-10b, activating the STAT3 pathway [90]
9.	CRNDE	Intergenic *chr16q12.2 (54,844,554-54,931,354)*	Promotes cell migration, invasion, and proliferation	Acts as a ceRNA against miR-423-5p, resulting in its downregulation [91]
			Confers cisplatin resistance	Activation of the SRSF1/TIA1 signaling pathway [92]
10.	DANCR	Intergenic *chr4q12 (52,712,257-52,797,688)*	Promotes cell proliferation and migration	Negative regulation of TGF- β by acting as a ceRNA for miR-214 [93]
11.	DUXAP10	Pseudogene *chr14q11.2 (19,294,785-19,337,674)*	Promotes cell proliferation	Increased expression of DUXAP10 positively regulates the proliferation of OC cells [94]
12.	EBIC	Pseudogene *chr16q23.1 (74,667,504-74,668,903)*	Promotes cell proliferation, invasion and migration. Confers cisplatin resistance	Activation of the Wnt/β-catenin pathway [95]
13.	ElncRNA1	Sense *chr1q32.1 (204,141,404-204,143,396)*	Oncogenic role in overall EOC progression	E2 (estrogen) transcriptionally induces ElncRNA1, which modulates cyclin D1-CDK4/6 [96]
14.	FLVCR1-AS1	Antisense *chr1q32.3 (212,851,961-212,858,309)*	Promotes EMT	Acts as a ceRNA to miR-513, upregulating YAP1 expression [97]
15.	H19	Intergenic *chr11p15.5 (1,995,129-2,004,552)*	Promotes cisplatin resistance	Glutathione metabolism [98]
16.	HAS2-AS1	Antisense *chr8q24.13 (121,639,292-122,111,171)*	Accelerates EOC tumorigenesis and facilitates invasion and proliferation	HAS2-AS1, induced by CREB1, sequesters miR-466, thus positively regulating the *RUNX2* gene [99]
17.	HCG18	Antisense *chr6p22.1 (30,221,090-30,327,401)*	Cell proliferation and migration	Acts as a ceRNA for miR-29a/b, downregulating TRAF4/5 and activating the NF-κB pathway [100]
18.	HOST2	Intergenic *chr10q23.1 (84,153,176-84,172,947)*	Promotes cell proliferation, migration, and invasion	Activation of the JAK2/STAT3 pathway [101]
19.	HOTAIR	Antisense *chr12q13.13 (53,962,188-53,975,055)*	Confers cisplatin resistance	Regulates Her2 expression by acting as a ceRNA against miR-138-5p [102]
20.	HOXA11-AS	Antisense *chr7p15.2 (27,184,507-27,189,298)*	Confers cisplatin resistance	Inhibits intracellular autophagy and cell cycle arrest [103]
21.	HOXD-AS1	Antisense *chr2q31.1 (176,164,050-176,189,421)*	Regulates cell migration, invasion, and EMT in EOC	Elevated HOXD-AS1 leads to increased levels of PIK3R3 by sequestering miR-186-5p (acting as a ceRNA) [104]
			Promotes cell proliferation, migration, and invasion and EMT in EOC cells	Activates the Wnt/β-catenin pathway by sequestering miR-133a-3p [105]
			Positively regulates proliferation, migration, and invasion in OC cells	HOXD-AS1 mediates this effect partially through the miR-608/FZD4 axis [106]
22.	HULC	Intergenic *chr6p24.3 (8,435,542-9,294,133)*	Confers paclitaxel resistance	Acts as a ceRNA against miR-199a-3p, upregulating the expression of ITGB8 [107]
23.	KCNQ1OT1	Antisense *chr11p15.5 (2,424,025-2,700,003)*	Enhances cell growth, migration, and invasion and inhibits cell apoptosis	Positively regulates LCN2 expression by repressing miR-212-3p [108]
24.	LINC-ROR	Intergenic *chr18q21.31 (57,054,557-57,072,314)*	Promotes EMT	Suppresses miR-145, promoting the expression of FLNB [109]
25.	LINC00152	Intergenic *chr2p11.2 (87,454,780-87,636,740)*	Confers cisplatin resistance in COC1/DDP cells	Modulates apoptosis and the expression of MDR1, GSTn, and MRP1 [110]
			Increased levels facilitate invasion and tumor proliferation in EOC	Prevents the ubiquitination of Bcl6 by binding to its Ser 333/Ser 343 site [111]
			Mediates cell proliferation and survival in OC	Affects MCL1-dependent mitochondrial apoptosis and acts as a ceRNA of miR-125b [112]
			Regulates cell cycle and cell proliferation in EOC cells	Modulates the tumor necrosis factor (TNF) signaling pathway [113]
26.	LINC00184	Intergenic *chr1q42.3 (234,609,295-234,686,426)*	Promotes cellular proliferation and confers cisplatin resistance	Promotes CNTN1 expression by acting as a ceRNA towards miR-1305 [114]
27.	LINC00319	Intergenic *chr21q22.3 (43,427,511-43,470,515)*	Facilitates proliferation, migration, invasion, and tumor growth	Upregulates NACC1 by sequestering miR-423-5p [115]
28.	LINC00511	Intergenic *chr17q24.3 (72,221,072-72,640,472)*	Promotes cell proliferation and invasion	Acts as a ceRNA against miR-424-5p and miR-370-5p which are responsible for anti-tumor effects [116]
29.	LINC00665	Intergenic *chr19q13.12 (36,259,540-36,332,581)*	Promotes tumor progression	Regulates the miRNA-34a-5p/E2F3 axis [117]
			Facilitates cancer cell proliferation and inhibits apoptosis	Upregulates FHDC1 by sequestering miR-181a-5p [118]
			Promotes cancer cell proliferation and migration	Positively regulates KLF5 via sponging miR-148b-3p [119]
30.	LINC00857	Intergenic *chr10q22.3 (80,206,672-80,235,950)*	Modulates OC progression and glycolysis	Regulates the Hippo signaling pathway through the miR-486-5p/YAP1 axis [120]
			Reduces the proliferative, invasive, and migratory capacity of OC cells and facilitates cell apoptosis	Reduces YAP-TEAD expression via the Hippo signaling pathway [121]
31.	LINC00858	Intergenic *chr10q23.1 (84,267,747-84,296,974)*	Contributes to the metastatic nature of OC	Acts as a ceRNA towards miR-134-5p, upregulating RAD18 expression [122]
32.	LINC00958	Intergenic *chr11p15.3 (12,928,291-12,989,650)*	STAT1-induced overexpression promotes overall EOC progression (proliferation, invasion, and migration)	Epigenetic modulation of the Wnt/β-catenin pathway [123]
33.	LINC00968	Intergenic *chr8q12.1 (56,493,948-56,560,407)*	Accelerates EOC progression	Arrests the cell cycle in the G1 phase by inhibiting the MAPK and PI3K/Akt/mTOR pathways [124]
34.	LINC01503	Intergenic *chr9q34.11 (129,320,968-129,359,711)*	Contributes to carboplatin resistance in OC	Upregulates PD-L1 levels by sequestering miR-766-5p [125]
35.	lncARSR	Intergenic *chr9q21.31 (79,505,801-79,571,041)*	Enhances EOC cells’ proliferative and invasive property	Upregulates β-catenin and ZEB1/2 via association with HuR and the miR-200 family, respectively [126]
36.	lncBRM	Intergenic *chr5q11.2 (57,570,341-57,629,629)*	Facilitates migration, invasion, and proliferation in OC cells	Upregulates SOX4 via sequestering miR-204 [127]
37.	MALAT1	Intergenic *chr11q13.1 (65,265,209-65,273,987)*	Induces cell proliferation, migration and EMT transition.	Activation of the PI3K/AKT pathway [128]
38.	MIAT	Intergenic *chr22q12.1 (26,646,411-26,676,478)*	Promotes EMT, migration, invasion, and proliferation	Acts as a ceRNA, resulting in suppression [129]
39.	MIF-AS1	Antisense *chr22q11.23 (23,893,709-23,902,114)*	Promotes cell proliferation, migration, and invasion	Acts as a ceRNA to miR-NA-31-5p, downregulating PLCB1 expression [130]
40.	MNX1-AS1	Antisense *chr7q36.3 (157,007,750-157,053,772)*	Promotes overall OC carcinogenesis	Upregulates SOX12 by repressing miR-744-5p [131]
41.	NEAT1	Intergenic *chr11q13.1 (65,416,581-65,450,093)*	Promotes cell proliferation and migration	Acts as ceRNA binding to let-7g promoting MEST and inhibiting ATGL expression [132]
			Confers cisplatin resistance	Regulates the expression of PARP1 and acts as a ceRNA against miR-770-5p [133]
42.	Lnc-OC1	Antisense *chr8q24.3 (142,688,218-142,727,056)*	Promotes cell proliferation and migration	Acts as a ceRNA to miR-34a and miR-34c which regulates tumorigenesis [134]
43.	PVT1	Intergenic *chr8q24.21 (127,794,513-128,188,211)*	Promotes cell migration and survival	Activation of YAP1-mediated tumorigenesis [135]
44.	SNHG1	Intergenic *chr11q12.3 (62,851,833-62,856,444)*	Promotes proliferation and migration in EOC	Activates downstream effectors of the Wnt/β-catenin pathway [136]
			Facilitates migration and invasion of OC cells	Modulates via the SNHG1/miR-454/ZEB1 axis [137]
			Modulates chemoresistance in SOC cells and patients (paclitaxel)	Functions as a ceRNA for miR-216b-5p in conferring paclitaxel resistance in OC [138]
45.	SNHG25	Antisense *chr17q23.3 (64,142,533-64,147,434)*	Promotes overall EOC progression	Positively regulates COMP (cartilage oligomeric matrix protein) contributing to the more invasive nature of the tumor [139]
46.	SOX2OT	Sense *chr3q26.33 (180,989,510-181,836,880)*	Facilitates OC progression	SOX2-OT contributed to OC malignancy through the miR-181b-5p/SCD1 axis [140]
47.	SRA	Intergenic *chr8p23.1 (10433672-10438312)*	Facilitates cell proliferation, migration, and tumor invasion	Acts via EMT and the NOTCH signaling pathway [141]
48.	TP73-AS1	Antisense *chr1p36.32 (3,735,984-3,747,373)*	Contributes to EOC carcinogenesis	Epigenetically suppresses p21 via trimethylation of H3K27 by recruiting EZH2 [142]
			Positively regulates tumor growth and metastasis and facilitates overall OC progression	Increased expression of TP73-AS1 enhances levels of MMP2 and MMP9 [143]
			Promotes proliferation and overall OC progression	Negatively regulates cellular apoptosis and the cell cycle [144]
49.	TPT1-AS1	Antisense *chr13q14.13 (45,341,344-45,417,975)*	Contributes to EOC tumor development and metastasis and inhibits cellular adhesion	Induces TPT1 expression and activates the PI3K/AKT pathway [145]
50.	TUG1	Intergenic *chr22q12.2 (30,969,245-30,979,395)*	Facilitates angiogenesis of endothelial cells in OC cells	Regulates LRG1 secretion levels partially via the TGF-β pathway [146]
			Promotes OC cell proliferation and malignancy	Acts as a ceRNA for miR-1299, thus positively regulating NOTCH3 expression levels [147]
			Affects OC progression and carcinogenesis	Works as an interacting component of the miR-582-3p/AKT/mTOR axis [148]
			Contributes to stemness, proliferation, and invasion in OC cells	TUG1 sequesters miR-186-5p to release ZEB1 [149]
			Confers autophagy-associated paclitaxel resistance in OC cells	Sequesters miR-29b-3p and consequently mediates paclitaxel resistance via autophagy induction [150]
51.	UCA1	Intergenic *chr19p13.12 (14,939,433-16,638,095)*	Confers cisplatin resistance	Acts as ceRNA to miR-27a-5p, regulating the expression of UBE2N [151] Acts as a ceRNA for miR-143, upregulating FOSL2 expression [152]
			Promotes proliferation, invasive migration, and therapy resistance	Sequesters a panel of the let-7 family of miRNAs, negatively regulating their tumor-suppressive roles [63]
52.	ZFAS1	Antisense *chr20p13.13 (49,276,738-49,361,1)*	Promotes cell proliferation and metastasis	Sequesters tumor-suppressive roles of miR-548e [153]
			Confers cisplatin resistance	Suppresses the expression of let-7a further elevating BCL-XL/S levels [153]

**Table 2:** LncRNAs that act as tumor suppressors in ovarian cancer.

	LncRNA	Position/Chromosomal Location	Function	Mechanism
1.	ADAMTS9-AS2	Antisense *chr3p14.1 (64,684,719-65,064,831)*	Inhibits cell proliferation and invasion	Acts as a ceRNA against miR-182-5p modulating the FOXF2 pathway [154]
2.	SLC7A11-AS1	Antisense *chr4q28.3 (138,027,409-138,191,769)*	Reduced SLC7A11-AS1 promotes EOC progression	SLC7A11-AS1 mainly deregulates the *SLC7A11* gene to suppress EOC progression [155]
3.	CASC2	Intergenic *chr10q26.11 (118,046,278-118,216,096)*	Inhibits migration, invasion, and proliferation	Reduced expression can be linked with poor prognosis in patient samples [156]
4.	DUXAP8	Pseudogene *chr22q11.1 (15,784,954-15,827,434)*	Regulate the proliferation and apoptosis of OC cells	Mediates YAP1 regulation via the suppression of miR-590-5p [157]
5.	FER1L4	Pseudogene *chr20q11.22 (35,558,737-35,607,562)*	Higher levels of FER1L4 facilitate paclitaxel sensitivity of OC cells	Suppresses paclitaxel resistance via inhibition of the MAPK pathway [158]
6.	GAS5	Antisense *chr1q25.1 (173,851,284-173,869,045)*	Inhibition of cell proliferation, migration, and invasion	Activation of the AKT/PTEN pathway by sequestering miR-96-5p [159]
7.	LIFR-AS1	Antisense *chr5p13.1 (38,556,762-38,719,004)*	Deregulation in OC cells and subsequent patients correlates to poor prognosis and increased carcinogenesis	Overexpression of LIFR-AS1 is associated with decreased invasion, migration, proliferation, and viability in SOC cells [160]
8.	LINC-PINT	Intergenic *chr7q32.3 (130,790,882-131,190,429)*	Inhibits cell migration, invasion, EMT, and proliferation and promotes cellular apoptosis (acts as a tumor suppressor)	Sequesters miR-374a-5p (acts as an oncogene) [161]
9.	LINC00641	Intergenic *chr14q11.2 (21,199,769-21,206,900)*	Suppresses the oncogenic role of miR-320a	Acts as a ceRNA for miR-320a which promotes cell migration and invasion [162]
10.	MAGI2-AS3	Antisense *chr7q21.11 (79,452,174-79,471,961)*	Suppresses the oncogenic role of miRNAs	Sequesters miR-15-5p, miR-374a-5p, and miR-374b-5p [163]
11.	MEG3	Intergenic *chr14q32.2 (100,779,206-100,861,031)*	Inhibits cellular proliferation and metastasis	Acts as a ceRNA against miR-885-5p increasing VASH1 expression [164]
12.	NBAT-1	Intergenic *chr6p22.3 (22,124,464-22,222,644)*	Suppresses tumorigenesis	Mediates its effect by targeting the AKT and ERK pathway [165]
13.	RP11-190D6.2	Antisense *chr16q23.1 (78,219,525-78,242,767)*	Low levels of RP11-190D6.2 associates with increased proliferative, invasive, and migratory properties in EOC	RP11-190D6.2 acts like a tumor suppressor where it confers its effects partly by regulating the expression of the gene *WWOX* [166]
14.	SDCBP2-AS1	Antisense *chr20p13 (1,273,409-1,386,950)*	Inhibits cell migration and invasion and increases the apoptotic rate	Sequesters miR-100-5p, upregulating its expression and downregulating EPDR1 expression [167]
15.	TUSC7	Intergenic *chr3p13.31 (116,642,614-116,723,581)*	Low levels of TUSC7 mediate proliferation, migration, and invasion in OC cells	Regulates the GSK3β/β-catenin pathway through the sponging of miR-616-5p [168]
16.	XIST	Intergenic *chrXq13.2 (73,817,774-73,852,753)*	Reduces tumor growth by inducing apoptosis	Sequesters against miR-106a [169]

## Abbreviations

The following abbreviations are commonly used in this manuscript:

OC	Ovarian Cancer
lncRNA	Long Non-Coding RNA
lincRNA	Long Intergenic Non-Coding RNA
ceRNA	Competing Endogenous RNA
miRNA	MicroRNA
piRNA	Piwi-Interacting RNA
snRNA	Small Nuclear RNA
circRNA	Circular RNA
siRNAs	Small Interfering RNAs
shRNA	Short Hairpin RNA
ASO	Antisense Oligonucleotide
RNA-seq	RNA Sequencing
CAF	Cancer-Associated Fibroblasts
CSC	Cancer Stem Cells
TME	Tumor Microenvironment
EMT	Epithelial–Mesenchymal Transition
